# Influence of electrode potential, pH and NAD^+^ concentration on the electrochemical NADH regeneration

**DOI:** 10.1038/s41598-022-20508-w

**Published:** 2022-09-30

**Authors:** Emad Aamer, Jorg Thöming, Michael Baune, Nicholas Reimer, Ralf Dringen, Manuela Romero, Ingmar Bösing

**Affiliations:** 1 Chemical Process Engineering Group (CVT), Leobener Strasse 6, 28334 Bremen, Germany; 2 Centre for Biomolecular Interactions Bremen (CBIB), Postbox 330 440, 28334 Bremen, Germany; 3 Center for Environmental Research and Sustainable Technology (UFT), Postbox 330 440, 28334 Bremen, Germany; 4 University of Bremen, Bremen, Germany; 5 MAPEX Center for Materials and Processing, Bremen, Germany; 6 University of Applied Science Bremerhaven, Bremerhaven, Germany

**Keywords:** Electrocatalysis, Biocatalysis, Electrochemistry

## Abstract

Electrochemical NAD^+^ reduction is a promising method to regenerate NADH for enzymatic reactions. Many different electrocatalysts have been tested in the search for high yields of the 1,4-isomer of NADH, the active NADH, but aside from electrode material, other system parameters such as pH, electrode potential and educt concentration also play a role in NADH regeneration. The effect of these last three parameters and the mechanisms behind their influence on NADH regeneration was systematically studied and presented in this paper. With percentages of active NADH ranging from 10 to 70% and faradaic efficiencies between 1 and 30%, it is clear that all three system parameters drastically affect the reaction outcome. As a proof of principle, the NAD+ reduction in the presence of pyruvate and lactate dehydrogenase was performed. It could be shown that the electrochemical NADH regeneration can also be done successfully in parallel to enzymatically usage of the regenerated cofactor.

## Introduction

Nicotinamide adenine dinucleotide (NADH) is an enzymatic cofactor that plays an essential role in many biochemical reactions^1^. Enzymatic reactions catalysed by many dehydrogenases rely on NADH as a cofactor for electrons and hydrogen shuttle. In nature, NADH is found in all living cellular organisms and is oxidised during cellular respiration^2^. Industrially it is used in the preparation of organic chiral compounds^3^. It can also be used in bioremediation for the aerobic transformation of chromium (VI), which is toxic, to chromium (III)^4,5^. There are numerous other uses of NADH which makes it an extremely important compound^6^. However, the industrial use of NADH is limited due to its high cost and low availability. Therefore, it is important to regenerate NADH from its oxidised form NAD^+^ to make its industrial use feasible^7^. Several methods of NADH regeneration have been tested so far including chemical, photochemical, biological, and electrochemical processes^8^. Among the electrochemical processes, mediated reduction of NAD^+^ has shown a high selectivity towards the enzymatically active 1,4-NADH. Steckhan and his colleagues discovered Cp*Rh(bpy)Cl^-^ as catalytic mediator^9^. Recent work has used this compound to functionalize electrodes^10–12^ or as an electron mediator in the electrolyte^13,14^. Furthermore, biologically based mediators such as diaphorase^15^ and enzyme cascades immobilized in an Indium Tin Oxide Titanium electrode^16^ have shown high selectivity towards active 1,4NADH. Nonetheless the electrochemical process for direct regeneration of NADH remains of great importance as the operational costs are low, chemical reducing agents or mediators are unnecessary, the reaction can be monitored and controlled easily by applying a potential and measuring current, and operational windows can be found, to reduce by-products during the synthesis, that must be later separated from the product itself^17^.

The electrochemical reduction of NAD^+^ to NADH proceeds in two steps^2,18^:

1 $${\text{Step}}1:{\text{NAD}}^{ + } + {\text{ e}}^{ - } { } \to {\text{NAD}}^{ \bullet }$$

2 $${\text{Step }}2{\text{a}}:{\text{NAD}}^{ \bullet } + {\text{ e}}^{ - } + {\text{ H}}^{ + } { } \to {\text{NADH}}$$

3 $${\text{Step }}2{\text{b}}:{\text{NAD}}^{ \bullet } + {\text{NAD}}^{ \bullet } { } \to {\text{NAD}}_{2}$$

The electrochemical reduction of NAD^+^ on bare metal electrodes at significantly high cathodic overpotentials takes place through one-electron transfer (step 1), which can be followed by a rapid reaction of the NAD^•^ radicals to form the inactive dimer NAD_2_^17,19^ (step 2b). The NADH regeneration-reaction crucial step consists of a second electron transfer followed by protonation (step 2a). Reaction step 2a can not only yield the desired active 1,4-NADH but also enzymatically inactive isomers 1,2-NADH and 1,6-NADH. This reaction step is slow compared to reaction step 2b which is mainly why the direct electrochemical regeneration of NADH on bare metal electrodes often yields high amounts of the enzymatically inactive dimer NAD_2_^2,20–22^. In another study^23^, Jaegfeldt observed that the second electron transfer occurs only at more (− 1.7 V) cathodic potentials compared to the first (− 1.1 V).

It could already be shown that the choice of the electro-catalysts for the electrochemical NADH regeneration plays an important role: on bare copper electrodes, Barin et al. achieved around 50% and on copper foam around 80% active 1,4-NADH^20^, while Damian could regenerate up to 80% active NADH on bare gold electrodes^18^. The electrochemical regeneration of NADH on titanium^2^ or modified glassy carbon electrodes^7^ was reported to yield up to 98% active NADH. Unfortunately, no information on the faradaic efficiency and the mole yield was given.

Not only the catalyst material plays a significant role in the NADH regeneration but also the electrode potential. As mentioned above, Jaegfeldt reported more negative potentials necessary for the second electron transfer step, which favours reaction step 2a and the protonation. On the other hand, the hydrogen evolution reaction (HER), which can be a competing reaction to the NAD^+^ reduction reaction, is also favoured at more cathodic potentials. It can be seen from the reaction equation that the reaction kinetics of step 2a depends upon the availability of H^+^ on the reaction site. If the availability of H^+^ on the surface of the electrode is decreased by consumption due to HER, it might diminish the overall NADH formed.

Therefore, it is not surprising that many authors found a dependency of the ratio of active NADH on the electrode potential. Ali et al. reported surprisingly high amounts of 1,4-NADH yield (98%) at a high negative potential of − 1.8 V vs Ag|AgCl|KCl_3M_^24^ with glassy carbon electrodes (our own experiments could not reproduce this yield). Contrary to their observations, Jaegfeldt et al. reported around 50% 1,4-NADH, 30% 1,6-NADH and 20% dimers at − 1.7 V vs Ag|AgCl|KCl_3M_^23^. Damian reported a decreasing yield of active NADH with increasing electrode potential^18,19^ and explained this observation with the higher amount of NAD^•^ radicals due to the enhanced reaction kinetics and transport at more cathodic potentials favouring dimerization. Barin et al. showed an initial increment in the yield of active NADH with decreasing potential and with further decreasing potential eventually a decreased yield of active NADH^21^.

Unfortunately, most of the studies do not discuss the faradaic efficiency of the process or quantify the concentrations of the regenerated active NADH, even though both are relevant parameters to evaluate the applicability of the process. Furthermore, many studies investigate the electrochemical NADH regeneration in phosphate buffer, even though it has been shown that NADH is unstable in this buffer solution^25–29^.

An overview of the current literature and the reaction scheme suggests that there are (next to the catalysts material) at least three system parameters that can affect the yield of active NADH: the electrode potential, the availability of H^+^, and the concentration of NAD^•^ radicals at the electrode, which is (among others) determined by the NAD^+^ concentration and the kinetic of the first electron transfer (Reaction 1). The latter, which is favoured by a high concentration of NAD^+^ or a high material transport to the electrode (caused by—among others—a high cathodic potential), might enhance the dimerization (as can be seen from reaction step 2b). The availability of H^+^ is essential for the protonation of the radicals (reaction step 2a), while the electrode potential controls the reaction kinetics of the NADH regeneration and the HER, which in turn might also influence the concentration of NAD^•^ radicals at the electrode surface and the H^+^ availability.

In the study presented here, these assumptions have been tested by systematically changing the solution’s pH and NAD^+^ concentration while studying the yield of active NADH for different electrode potentials on a bare copper electrode. Furthermore, the faradaic efficiency of the process has been calculated and the molar yield is presented as well as the dependency on all three system parameters—electrode potential, pH and substrate concentration, on the electrochemical NADH regeneration. The results presented in this manuscript deliver a deeper understanding of the direct mechanism of NADH regeneration either on bare or on pre-treated electrodes.

Additionally, we propose that it can be advantageous to combine the electrochemical regeneration of NADH with enzyme-catalysed reactions, using the regenerated cofactor directly to produce valuable chemical compounds, e.g. for industrial use. This has been done for reducing reactions to facilitate the production of chemicals in an NADH-dependent manner, for example by reducing CO_2_ to formate with the formate dehydrogenase^30^. To test, if enzyme activity can be maintained under the applied potential and during the NADH regeneration, lactate dehydrogenase (LDH) and pyruvate are added to the reaction chamber in a proof of principle experiment.

## Experimental

All electrochemical measurements were carried out in a three-electrode H-cell containing 0.1 M Tris buffer at room temperature, where anodic and cathodic half cells were separated by a glass frit. A copper sheet (99.9% purity) of size 1.5 cm $\times$ 1.5 cm was used as the working electrode. Before each measurement, it was ground and polished with diamond paste (6 µm) and cleaned with ethanol and deionized water. An Ag/AgCl (3 mM KCl) electrode served as reference electrode while a platinum sheet electrode was used as counter electrode. The electrochemical cell was purged with nitrogen for at least 15 min before each measurement and the subsequent measurements were carried out in a nitrogen atmosphere. The potentiostat used was a Metrohm Autolab PGSTAT204 with the Nova software package. The Linear Sweep Measurements were performed with a scan rate of 0.1 V/s between 0 and − 1.5 V (vs. Ag/AgCl). All potentials in this paper refer to a Ag/AgCl electrode, where cathodic potentials are negatively drawn. The regeneration measurements were carried out at constant potentials for 1800s.

The determination of active NADH was done with an enzyme test involving lactate dehydrogenase (LDH), pyruvate, and consequent measurements of the extinction at 340 nm with a UV/VIS spectrometer.

The addition of pyruvate and LDH leads to the reaction of enzymatically active 1,4-NADH as follows:

$${\text{pyruvate}} + 1,4 - {\text{NADH }}\to ^{{\text{ LDH }}} {\text{ lactate}} + {\text{NAD}}^{ + }$$

Three possible products of the NAD^+^ reduction, 1,4-NADH, 1,6 NADH and NAD_2_ show extinction at 340 nm^8,19^ making it impossible to determine the percentage of active 1,4-NADH by UV/VIS measurements alone. By measuring the extinction after the NAD^+^ reduction (while the solution contains 1,4-NADH, 1,6-NADH and NAD_2_) and after the addition of LDH and pyruvate, when all 1,4-NADH is consumed by the reaction with pyruvate, and calculating the difference of both extinctions (before addition of LDH—including 1,4-NADH and after addition of LDH—without 1,4-NADH), the percentage of active NADH can be calculated. It should be mentioned that the addition of pyruvate also increases the extinction at 340 nm, which must be then subtracted from the results.

Enzymatic utilization of regenerated NADH was determined during electrochemical NADH regeneration under a potential of − 1.3 V at a solution pH of 7 with 0.75 mM NAD^+^, and 5 mM pyruvate with and without 2 U/mL LDH. The total volume in the reaction chamber was 15 mL. During regeneration, samples of 300 µL were taken from the reaction chamber at regular time intervals. Each measurement was repeated at least three times.

## Results and discussion

### Buffer system

Many research groups working on the electrochemical reduction of NAD^+^ have used phosphate as the buffer system^7,17,20–22^. This is not advisable, as it was shown that NADH is unstable in phosphate buffer^26–29^. An increased degeneration of NADH in combination with an enzyme assay to test the ratio of active NADH will lead to an overestimation of active NADH since the degeneration of (active and inactive) NADH and NAD_2_ due to the buffer system will lead to a decreasing signal in the UV/VIS at 340 nm.

**Figure 1:**
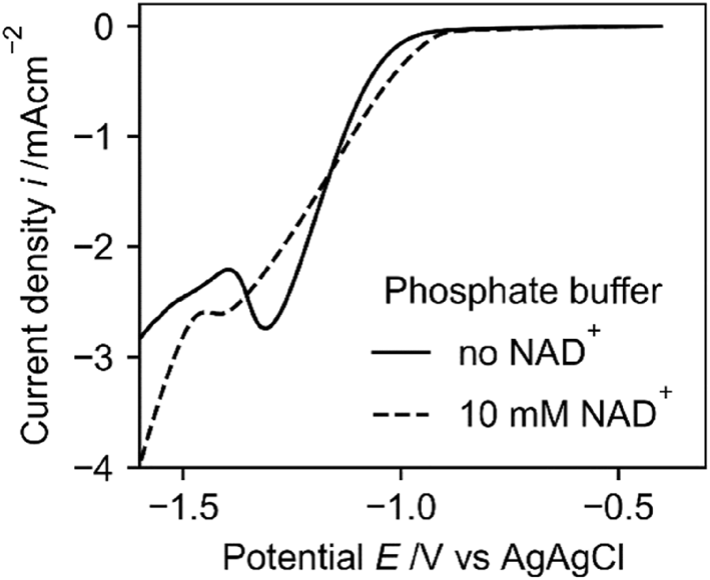
Linear potential sweep from − 0.4 to − 1.4 V with the scan rate of 0.1 V/s vs. Ag|AgCl|KCl3M in 0.1 M phosphate buffer pH 7 with and without NAD + at a copper electrode.

Furthermore, LSV measurements in phosphate buffer show a reduction peak due to the reduction of phosphate groups at the same potential as the NAD^+^ reduction occurs (Fig. 1), which will lead to a worse faradaic efficiency. As can be seen from the polarization measurements with NAD^+^ in phosphate buffer the reduction peak slightly shifts to more cathodic potentials and the peak current (attributed to the reduction of NAD^+^ and phosphate groups) decreases by addition of NAD^+^. This behaviour can be attributed to adsorption of NAD^+^ at the electrode surface, which blocks parts of the electrode for phosphate reduction reactions. The blocking depends on the electrode material and could also be observed at titanium electrodes^2^.

**Figure 2:**
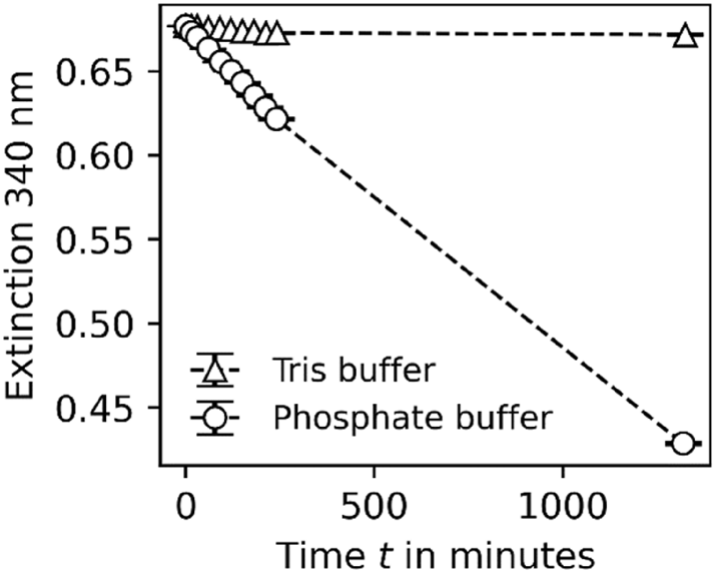
Extinction at 340 nm in the percentage of start extinction over time for 0.1 mM NADH in Tris buffer ($\Delta )$ and Phosphate buffer (O) (both pH 6.8).

To test if NADH shows better stability in Tris buffer, phosphate and Tris buffer solutions at different pH (6.8, 7.2, 7.6) values containing 1.0 mM NADH were prepared and the extinction at 340 nm was measured over time by UV/VIS measurements. While the extinction decreases drastically in phosphate buffer (nearly 60% of start extinction after 1300 min), the extinction remained constant in Tris buffer (Fig. 2), indicating better stability of NADH in Tris buffer over time.

### Reduction of NAD + 

**Figure 3:**
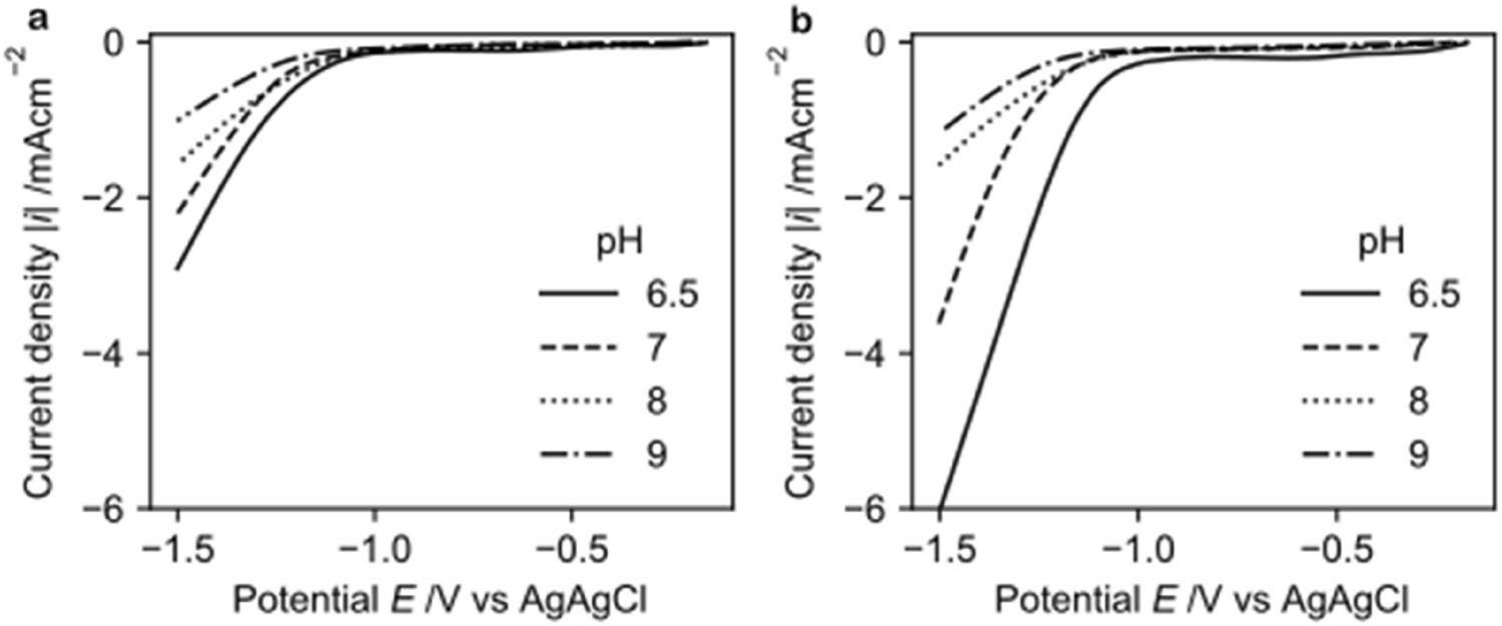
LSV taken using a copper electrode in 0.1 M Tris buffer flushed with N_2_ at pH 6.5, pH 7, pH 8 and pH 9 with a scan rate of 0.1 V/s. (**a**) without NAD^+^ (**b**) with 1.5 mM NAD^+^.

Linear sweep voltammetry (LSV) was performed to identify the electrochemical behaviour of NAD^+^ reduction at different pH values and to identify the potential where hydrogen evolution reaction starts. Figure 3a and b show the LSV taken on a copper electrode in the absence and the presence of NAD^+^, respectively. The LSV was performed in the potential region − 0 to − 1.5 V vs Ag/AgCl (3 M KCl). An increase in current density at higher cathodic potential is due to hydrogen evolution reaction (HER), which is kinetically favoured at higher cathodic potential, and at lower pH values as observed in Fig. 3a. The current density at a certain pH is lower (less cathodic) when NAD^+^ is not present in the electrolyte. However, when NAD^+^ is added to the electrolyte, a significant increase in current density results due to the reduction of NAD^+^. The reduction potential of NAD^+^ as seen from the increase of cathodic current was in agreement with prior research^7,19,20,31^. In the presence of NAD^+^, reduction starts at around − 0.9 V. However, Fig. 3 suggests that to achieve the NAD^+^ reduction at an appreciable rate, higher cathodic potentials are required.

### Influence of pH

The electrochemical behaviour of NAD^+^ at different potentials was previously studied^2,19^. It has been discussed that the yield of enzymatically active NADH depends upon the electrode potential and the electrode material^19^. Both, the electrode’s material and potential directly affect the amount of adsorbed hydrogen H_ads_ at the surface of the electrode which is available for NAD^•^ radicals to form NADH, and the hydrogen evolution reaction. However, the availability of H^+^ and thus the amount of adsorbed H_ads_ can also be affected by changing the pH of the electrolyte. Thus, if the above-stated hypothesis is true—the adsorbed H_ads_ affect the NADH regeneration—one should observe a dependency of active 1,4-NADH on the solution’s pH.

To study the influence of electrolyte’s pH several experiments were carried out in the pH range between 6.5 and 9.0 in a batch electrochemical cell using Cu as the working electrode. The percentage of enzymatically active 1,4-NADH formed was determined by an enzyme assay as described in the experimental section.

**Figure 4:**
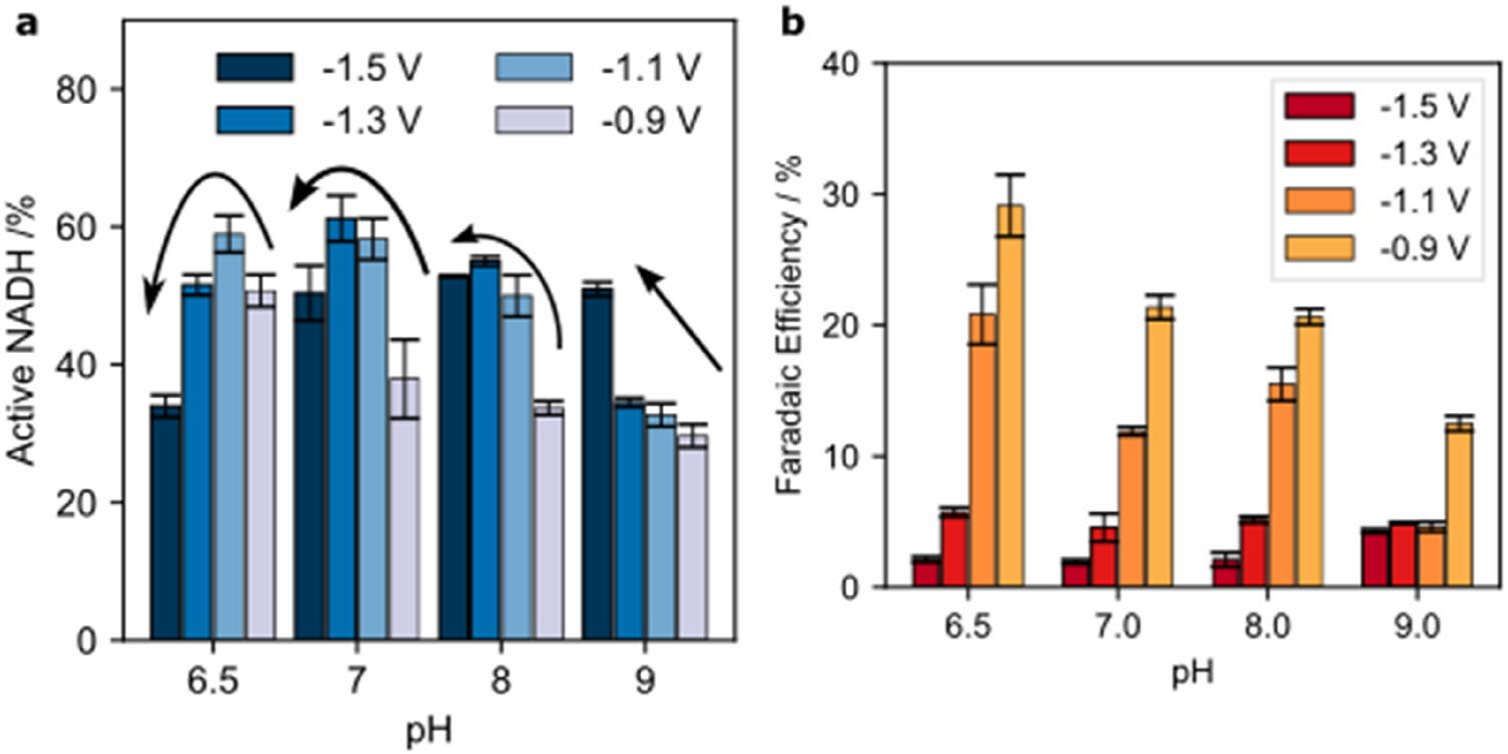
Influence of pH and electrode potential on the NADH regeneration. (**a**) Percentage of active 1,4-NADH produced with a copper electrode at potential (− 0.9 to − 1.5 V) vs. Ag|AgCl|KCl_3M_ and using 1.5 mM NAD^+^ in Tris buffer for 1800s. (**b**) Faradaic efficiency of the NADH regeneration.

The percentage of regenerated, enzymatically active 1,4-NADH strongly depends on the pH of the electrolyte as well as the electric potential (Fig. 4). At the highest pH (pH 9.0) increasing the cathodic potential from − 0.9 to − 1.5 V results in an increase of the average amount of active 1,4-NADH. At low cathodic potentials, the first electron transfer occurs drastically faster compared to the second electron transfer, thus NAD^•^ radicals form at the electrode surface and their dimerization is faster than the second electron transfer. Furthermore, at low cathodic potentials (and high pH values) the adsorption of hydrogen to the electrode surface by the Volmer reaction^32^ is very low, thus the availability of adsorbed hydrogen for the formation of NADH is limited. With increasing electrode potential, the reaction kinetics of the second electron transfer step and the hydrogen adsorption at the electrode surface increase. Therefore, with increasing cathodic potential the percentage of active 1,4-NADH increases at pH 9. It should be noted that the total amount of active NADH at pH 9 and reduction potentials of is very low (as can be seen below).

With decreasing pH and potential until − 1.1 V (in case of pH 6.5) or − 1.3 V (in case of pH 7 and pH 8) there is a visible increase of active NADH. But by further decreasing the potential a decrease in active NADH occurs. This is due to an increase in the HER, which limits the amount of H_ads_ necessary for the NADH regeneration. At lower pH and potential values, the reaction kinetics of the HER are faster than reaction step 2a.

As an overall trend from pH 9 to pH 7, with decreasing pH, an increasing percentage of active NADH can be seen. With further decreasing pH to 6.5 the percentage of active NADH decreases due to the accelerated HER. This observation is further supported by the course of the faradaic efficiency (FE) for the 1,4-NADH regeneration at different potentials and pH values (Fig. 4b): with increasing cathodic potential from − 0.9 to − 1.5 V the faradaic efficiency drastically drops for all pH values, due to the accelerated HER and due to a large number of electrons being used for the non-desirable HER and formation of NAD_2_. At − 1.5 V the highest faradaic efficiency can be observed for pH 9 (slowest HER) and the lowest FE can be observed at pH 6.5 (fastest HER). At an electrode potential of − 0.9 V, one can observe the lowest FE at pH 9 (lowest availability of H_ads_ and thus favoured dimerization), while the highest FE can be observed at pH 6.5 (high H_ads_ availability).

**Figure 5:**
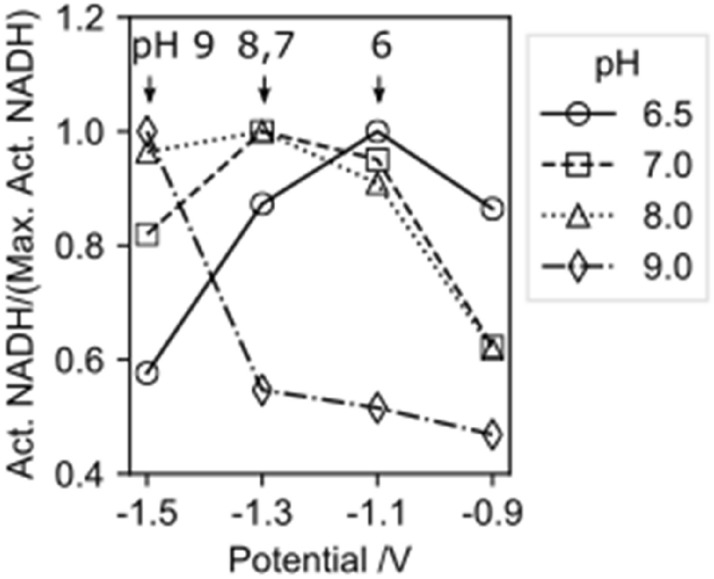
Normalised amount of active NADH over the electrode potential for different solution pHs. Arrows indicate the tipping potential for the different solution pHs. The normalised active NADH was calculated by dividing the active NADH by the maximum active NADH of each pH.

Due to the interaction of both competing reactions, HER and NADH regeneration, which are influenced by the electrode potential, for each pH value a specific potential depending peak of active NADH can be found (Fig. 5). This tipping potential—the potential at which the highest percentage of active 1,4-NADH can be found and at which a further decrease of electrode potential will lead to a worse yield of active NADH, is strongly pH-dependent. The higher the pH, the more cathodic the tipping potential. Accordingly, the most cathodic tipping potential (− 1.5 V) can be found for pH 9 and the least cathodic tipping potential (− 1.1 V) for pH 6.5.

**Figure 6:**
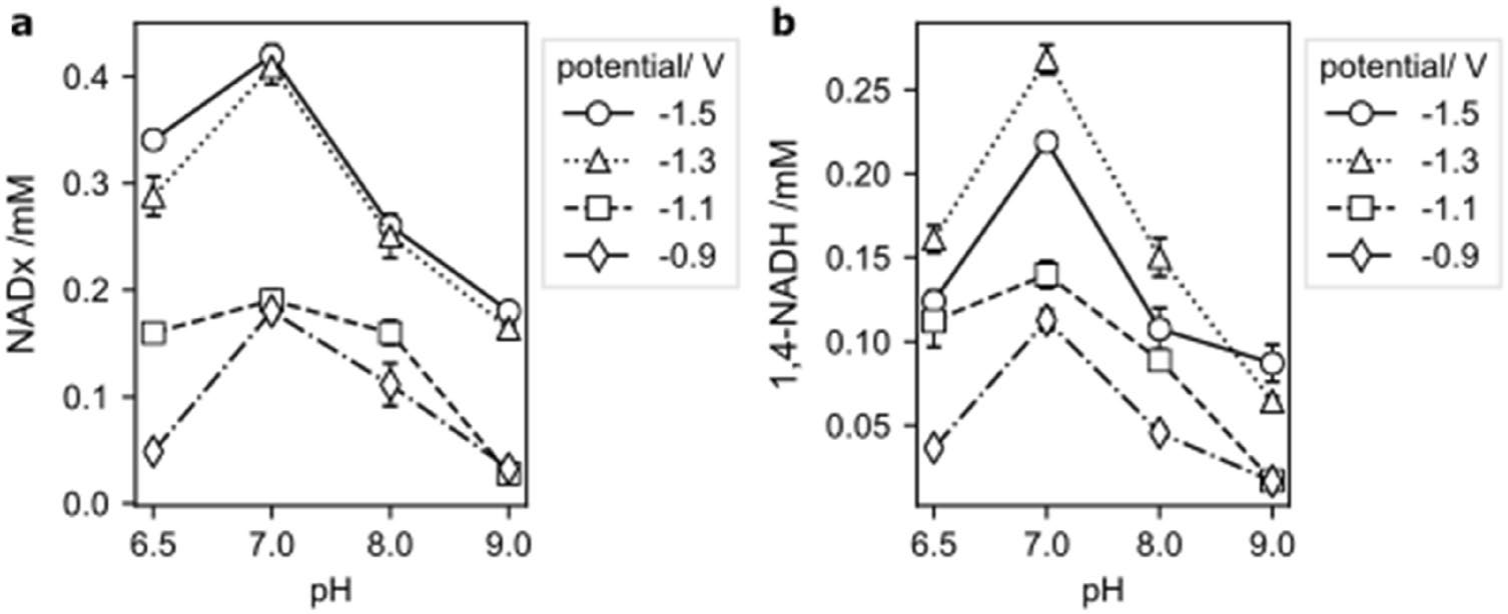
Moles of reaction products after 30 min of NAD^+^ reduction depending on the electrode potential for different pH. (**a**) NADx (NADH isomers and NAD_2_); (**b**) 1,4 NADH.

The total amount of NADx (all NADH isomers and NAD_2_ dimer) generated during 30 min of electrochemical reaction depends on pH as well as the electrode potential (Fig. 6a). Lowering the electrode potential from − 0.9 V to more cathodic potentials increases the number of moles generated at all different electrolyte pH values, due to the enhanced reaction kinetics. However, it is also shown that if the pH is increased, while the electrode potential is kept constant, the moles of NADx first increase from pH 6.5 to pH 7, and later decrease between pH 7.0 and pH 9.0. With decreasing pH, the HER is favoured over the NAD^+^ reduction, which leads to a drop of regenerated NADx and pH 7.0 seems to be the ideal combination of available H_ads_ and low HER.

The data obtained for the amounts of active 1,4-NADH formed within 30 min at different pH-values are similar to that observed for the sum of all NADx species (Fig. 6b). But in contrast to the overall amounts produced, the dependency on the electrode potential shows a difference. A maximum of active 1,4-NADH was observed at − 1.3 V, since more cathodic potentials (− 1.5 V) lead to faster Hydrogen Evolution Reaction and result in higher dimerization due to the lack of adsorbed H_ads_. At any given potential, pH 7.0 regenerated the maximum amount of 1,4-NADH. At lower pH than 7.0, HER reaction is favoured over reaction step 2a, while at pH values higher than 7.0 dimerization is kinetically favoured due to less availability of H^+^.

### Influence of NAD^+^ concentration

Damian et al. showed that increasing the concentration of NAD^+^ in the bulk solution increases the surface coverage of the NAD^+^ on the electrode rapidly^19^. Increasing the concentration further would level off indicating that the saturated monolayer NAD^+^ coverage is reached^18^. But this is only true for less cathodic potentials where the competing reactions are not taking place. However, if the cathodic potential is higher or near the NAD^+^ reduction potential, then the reduction reaction is faster than the adsorption of NAD^+^ molecules on the electrode surface^33^. Hence, the reduction of NAD^+^ will be diffusion controlled at higher cathodic potentials.

The more cathodic the potential, the faster the formation of radicals, which leads to a steep concentration gradient of NAD^+^ and thus, higher mass transport by diffusion towards the electrode’s surface. This may lead to an accumulation of NAD^•^ radicals on the electrode’s surface, which would lead to an accelerated formation of NAD_2_ dimers since reaction step 2b depends on the dimer concentration. If this—next to the accelerated HER and the depletion of adsorbed hydrogen—is a further reason for the decrease of active 1,4-NADH at high cathodic potentials, this must be a concentration-dependent phenomenon. As a consequence, NAD dimerization should be decreased with lower NAD^+^ concentration which should lead to a percental enrichment of NADx in active 1,4-NADH.

**Figure 7:**
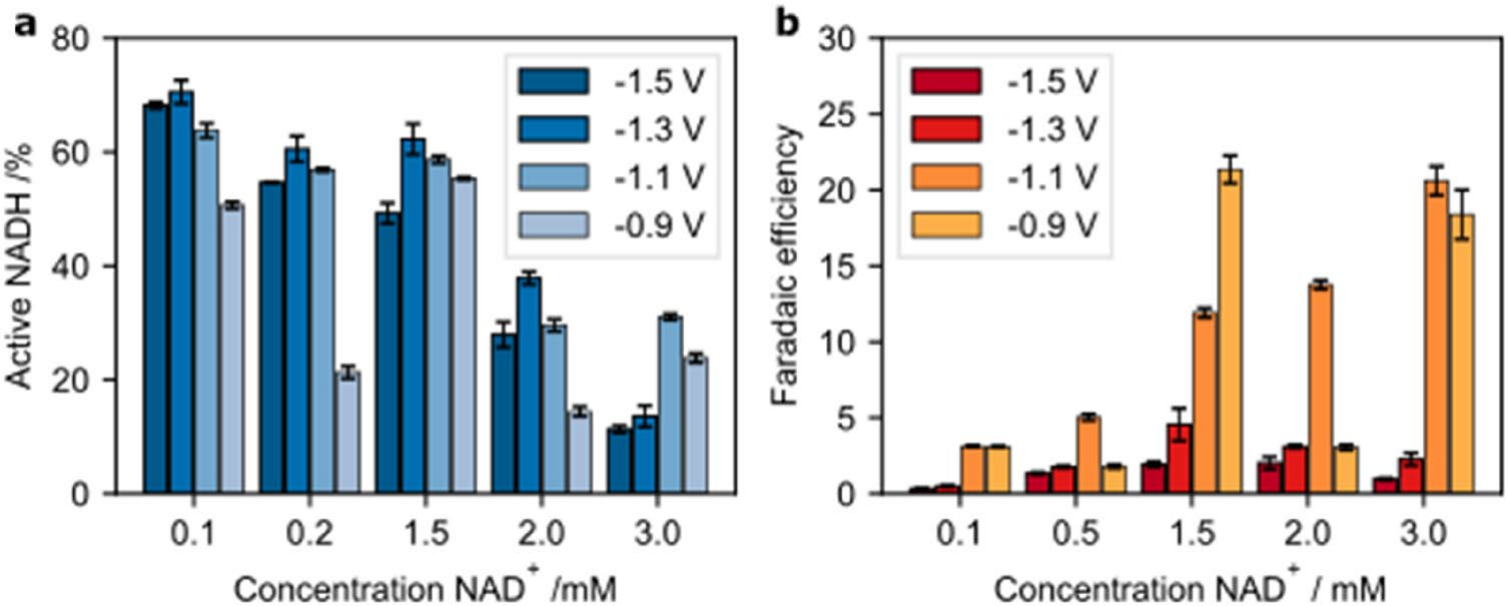
Concentration depending on NADH regeneration for different electrode potentials. (**a**) Percentage of active NADH depending on NAD^+^ concentration and electrode potential; (**b**) Faradaic efficiency of 1,4-NADH regeneration depending on NAD^+^ concentration and electrode potential.

By decreasing the NAD^+^ concentration from 3 mM stepwise down to 0.1 mM, the percentage of active 1,4-NADH increases significantly for all electrode potentials (Fig. 7a). The tipping potential—the potential at which a more cathodic potential will lead to a decreasing 1,4-NADH percentage—of the 3 mM solution is − 1.1 V whereas the tipping potential for the remaining concentrations is − 1.3 V. At the lowest concentration, 0.1 mM NAD^+^, the difference of active NADH between − 1.3 and − 1.5 V is smallest. These observations support the hypothesis that the reaction in step 2b, the dimerization, is a concentration-dependent reaction.

The effect of concentration on faradaic efficiency displays a less clear trend (Fig. 7b). The lowest concentration of NAD^+^ shows the lowest faradaic efficiency, which might be due to a low NAD^+^ concentration at the electrode’s surface and thus elevated HER compared to NAD^+^ reduction. In addition, only for a concentration of 1.5 mM, the FE decreases steadily with higher cathodic potentials. In the case of the remaining concentrations, the highest FE can be found at − 1.1 V, which might be due to the combination of relatively high 1,4-NADH yield and low HER.

### Enzymatic utilisation of produced NADH during regeneration

**Figure 8:**
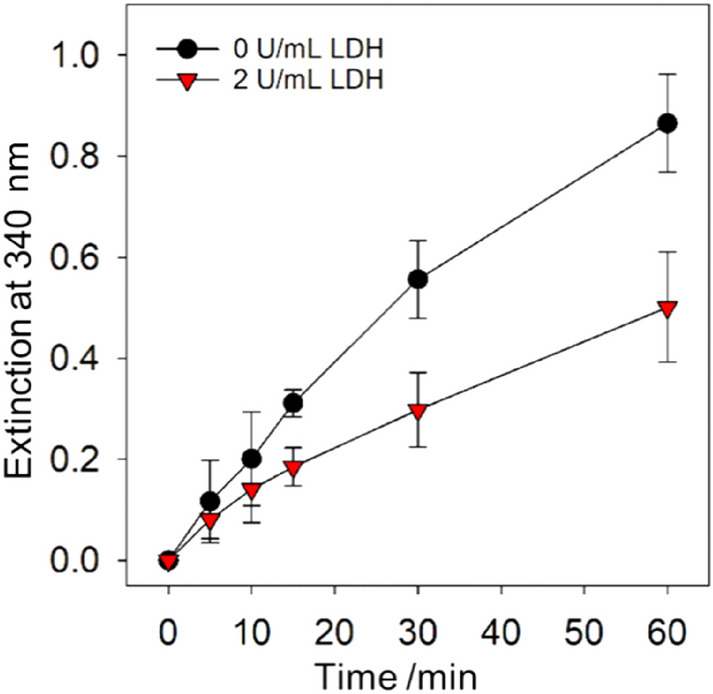
Extinction at 340 nm over time during electrochemical NADH regeneration from 0.1 M Tris buffer (pH 7) containing 0.75 mM NAD^+^ and 5 mM pyruvate at − 1.3 V, with (triangle) and without (circle) addition of 2 U/mL LDH.

To test if the optimised NADH regeneration conditions provided sufficient active 1,4-NADH to continuously allow an enzymatic reduction reaction, LDH was either added or not to the reaction chamber, containing 0.75 mM NAD^+^ and 5 mM pyruvate, before the electrochemical NADH regeneration at − 1.3 V was started. Samples were taken from the reaction chamber for different regeneration times, and their absorption at 340 nm was recorded (Fig. 8) after one hour 1.3% of the pyruvate was converted to lactate. Similar approaches of parallel NAD^+^ reduction and enzymatic reaction using the regenerated NADH have been performed with mediated NAD^+^ reduction by usage of a Rhodium complex^12,13^ or poly(neutral red)^34^.

During the NADH regeneration, a clear increase in absorption over time was observed for both scenarios, reactions with and without LDH. As expected, the absorbance values for LDH- and pyruvate-containing solutions were significantly lower, showing around 55% of the values for the solutions regenerated without the enzyme. This suggests that at least around 45% of the regenerated NADx is active 1,4-NADH (since it is consumed by the LDH). This proves that coupling the electrochemical regeneration at bare metal electrodes and enzymatic reaction in the same reaction chamber is possible.

An amount of 45% active NADH is lower compared to the 70% achieved under similar conditions without LDH and pyruvate, this can have different causes: Enzymatic reactions take time, and continuously electrochemically regenerated 1,4-NADH at the electrode take longer to couple with the LDH and form lactate. Additionally, LDH does not work optimally in the presence of the copper electrode. It has been shown in the literature that copper ions can reduce the activity of enzymes^26^. Finally, the applied voltage might have an impact on LDH’s activity and prevent it from converting all active 1,4-NADH to lactate.

This experiment was designed to provide proof of concept. We will further investigate whether and how the applied potential and ongoing electrochemical processes alter the catalytic behaviour of enzymes.

## Conclusion

Three different parameters influencing the electrochemical NADH regeneration and the yield of active 1,4-NADH from this reaction were tested. First, the solution’s pH was changed, which affected the availability of protons and adsorbed H_ads_ at the electrode’s surface, necessary for the formation of NADH from NAD^·^ radicals (reaction step 2a). The NAD^+^ concentration was changed, which affected the concentration of newly formed radicals at the electrode’s surface and thus the possibility of radical dimerization to NAD_2_ according to reaction step 2b. As for the third parameter, the electrode potential was changed for all tested pH values and concentrations. The electrode potential controls the reaction kinetics and delivers the energy for the first electron transfer (reaction step 1), which affects the NAD^·^ radical concentration and the second electron transfer (reaction step 2a) for the production of NADH. In addition, the potential controls the HER which affects the availability of protons and adsorbed hydrogen H_ads_.

Changing the pH of the solution shows a significant effect on the percentage of 1,4-NADH. The combination of electric potential and the solution’s pH controls the HER which affects the NADH regeneration. Thus, the tipping potential for maximum active NADH shifts with increasing pH to more cathodic potentials.

By lowering the NAD^+^ concentration a higher percentage of active NADH can be achieved. This is due to an overall lower NAD^·^ radical concentration at the electrode surface and thus, a less favoured dimerization.

Even on a bare metal electrode the range of active NADH could yield between approx. 10 and 70% and the faradaic efficiency between approx. 1 and 30% simply by adjusting different system parameters. The understanding of the parameters affecting the electrochemical NADH regeneration, presented here, will help to optimise conditions which may allow finally to bring the process from laboratory to application scale.

Lastly, LDH and pyruvate were added to the reaction chamber during the regeneration of NADH from NAD^+^. The data from this experimental setup suggests that the enzyme could use the 1,4-isomer formed continuously during the electrochemically NADH regeneration and catalyse the reaction converting pyruvate to lactate. Future experiments should further explore how to improve the yield of active 1,4-NADH during electrochemical NAD^+^ reduction in the presence of enzymes to enhance the online use of regenerated, active NADH for enzymatic reduction reactions.

## Data Availability

All data will be available on reasonable request from the corresponding author.
